# The relationship between health behaviors and quality of life: the mediating roles of activities of daily living and psychological distress

**DOI:** 10.3389/fpubh.2024.1398361

**Published:** 2024-05-28

**Authors:** Yuhang Wu, Jianqiang Xu, Yang Gao, Juan Zheng

**Affiliations:** School of Management, Xuzhou Medical University, Xuzhou, Jiangsu, China

**Keywords:** older adults, health behavior, activities of daily living, psychological distress, QOL

## Abstract

**Objective:**

The aim of this study is to examine the role of activities of daily living performance (ADLs) and psychological distress in mediating the process by which health behaviors affect QOL.

**Methods:**

A non-probabilistic study was conducted among 1,065 older adult people older than 60 years. Participants were assessed using the Barthel Index, Functional Activities Questionnaire (FAQ), Kessler Psychological Distress Scale (K10), Australian Active Survey, and EQ-VAS score. The SPSS22.0 software was used to analyze the differences in QOL scores among older adults with different demographic characteristics. Pearson correlation analysis was used to analyze the correlation between health behaviors, psychological distress, ADLs, and QOL. Amos23.0 software was used to construct structural equation model (SEM) to analyze the path of health behavior affecting QOL and the mediating role of BADLs, IADLs and psychological distress.

**Results:**

(1) The direct effect of health behaviors on QOL was not significant in the model; (2) ADLs had multiple mediating effects on the relationship between health behaviors and QOL, and the incidence of ADL limitation was negatively correlated with the reported QOL in the older adult; (3) Psychological distress had a significant mediating effect on the relationship between health behaviors and QOL.

**Conclusion:**

The results of this study elucidated the mechanisms of the correlation between health behaviors and QOL, and added to the existing literature. In addition, these mediating factors and indirect pathways have been identified as targets for intervention to improve the QOL of older adult individuals, which is important for achieving healthy aging.

## Introduction

Population aging is occurring worldwide (1). China is the country with the largest older adult population in the world (2). According to the United Nations “World Population Prospects” and China’s seventh national census, the proportion of the older adult population among the total population is 18.7%, and this proportion is expected to increase to 38.81% by 2050 (3, 4). With the acceleration of the aging process, the quality of life (QOL) of older adult individuals has attracted increasing attention from society. QOL is a comprehensive measure of the physical, psychological, environmental and social status of an individual or group (5, 6). As an important concept and goal of research and practice in the field of health and medicine, QOL is significant for assessing the physical and mental health of older adult individuals (5, 7). Studies have shown that health behaviors are an important factor affecting QOL in older adult individuals, and adopting positive health behaviors such as quitting smoking, avoiding excessive consumption of alcohol and engaging in long-term moderate physical exercise can improve physical and mental QOL (8, 9). In addition, the association between health behaviors and QOL in older adult individuals is affected by several factors. Previous studies have shown that the performance of activities of daily living (ADLs) and psychological distress, such as depression, anxiety and stress, affect QOL in older adult individuals (8, 10).

Decreased ADL ability and the presence of psychological distress are physical and mental health problems that manifest with age (11, 12). These problems are partly influenced by health behaviors (11, 13). Previous studies have shown that health behaviors are a combination of different behavior patterns and that health behaviors such as smoking, drinking and engaging in physical exercise can significantly affect changes in ADL ability and psychological distress (12–14). ADLs refer to the most basic and common activities that people carry out repeatedly every day to maintain survival and adapt to the living environment (15). Negative health behaviors, such as smoking, heavy alcohol consumption, and reduced physical activity, lead to loss of physical function and dependence on assistance in transferring, healthcare, tool use, communication, household work, and social cognition, as well as the inability to independently perform ADLs (16). While smoking cessation and active physical exercise can improve the ADL ability of older adult individuals, there is no strong evidence on the mechanism of alcohol abstinence in improving ADL ability in older adult individuals (8). Symptoms of depression, anxiety and stress are collectively referred to as psychological distress (15). Some studies have shown that health behaviors are closely related to psychological distress (13, 17), with smoking, alcohol consumption and reduced physical activity leading to increased psychological distress, anxiety, depression and perceived stress (13, 18). Quitting smoking, reducing alcohol intake and engaging in moderate physical activity can improve mental health (17, 19, 20). In addition, ADL ability is an indicator of a person’s functional state and has a significant impact on mental state (15). Previous studies have found that when older adults have limitations in performing ADLs independently, they are chronically dependent on others for help, lack confidence in solving problems, and suffer psychological distress such as depression, anxiety, and stress (21). Overall, health behaviors, ADL performance, psychological distress, and QOL are correlated, but few previous studies have reported on the mediators of the relationship between these variables.

**Figure 1:**
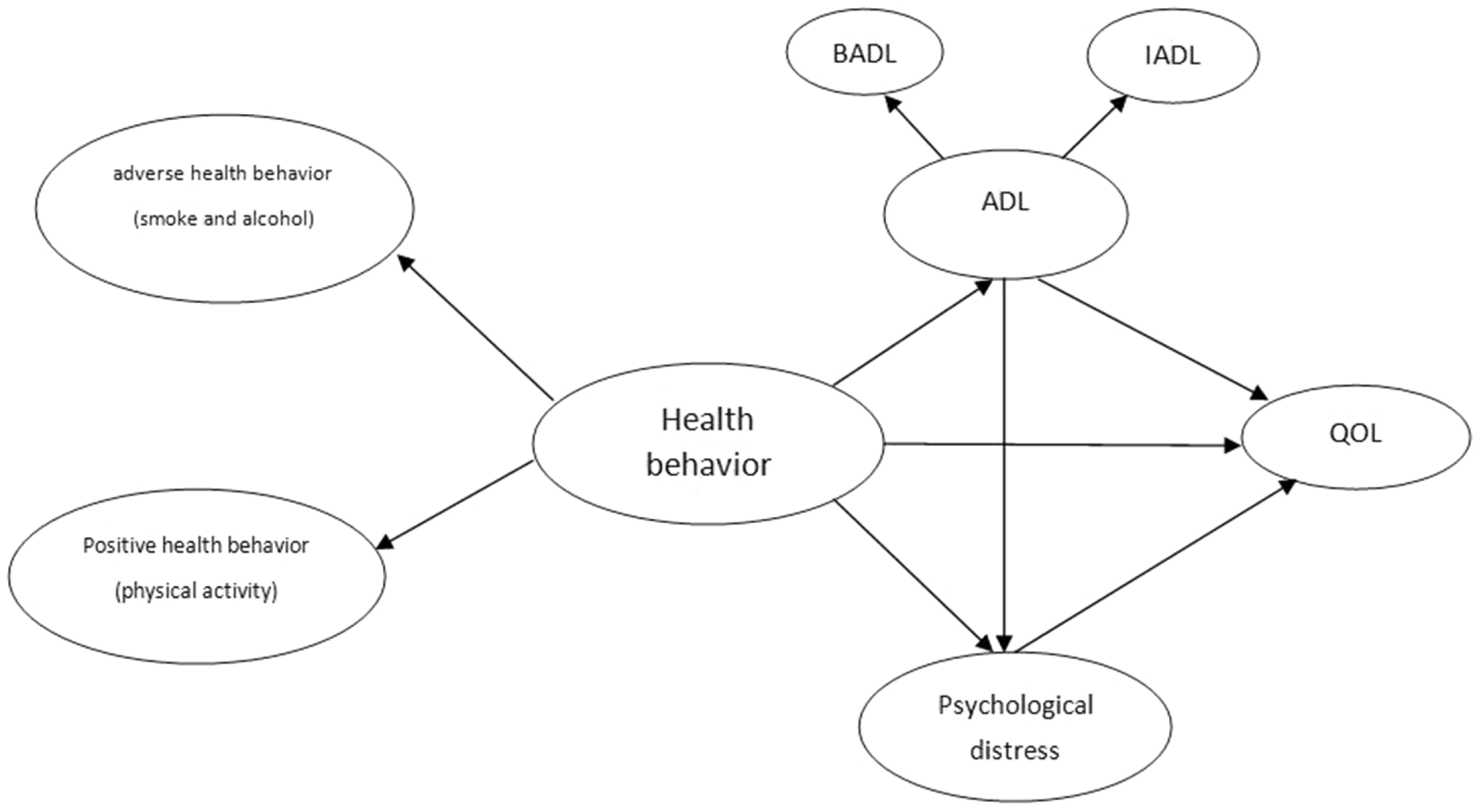
Hypothesized pathways from health behaviors to QOL in the conceptual model.

In summary, based on the above studies, it can be argued that ADL performance and psychological distress have multiple mediating effects on the relationship between health behaviors and QOL, as such effects also change with changes in health behaviors. However, the current research on the mechanism of the effect of health behaviors on the QOL of older adults is limited, and further research should be carried out. Therefore, exploring this mechanism from the perspective of ADL performance and psychological distress is of great significance for improving the QOL of older adult people. In summary, the aim of this study is to investigate the relationship between health behaviors and QOL, explore the role of ADL performance and psychological distress as mediators (see Figure 1 for the hypothesized model), and thus provide a theoretical basis and effective guidance for improving health behaviors and improving the QOL of older adults.

## Methods

### Study design and respondents

This non-probabilistic study was conducted between June 30 and August 28, 2022, in Xuzhou, China, with a quota sample of respondents that was representative of the regional population in terms of gender, age, household registration, marital status, education, smoking status, alcohol consumption and physical activity. A total of 1,065 respondents over 60 years old were recruited to complete an offline survey collecting data on (1) sociodemographic characteristics; (2) health behaviors; (3) ADL performance; (4) psychological distress; (5) QOL. The inclusion criteria for the survey respondents were (1) aged 60 or over; (2) long-term residence at the survey site for more than 6 months; (3) normal language expression and good communication skills; (4) voluntary acceptance of the survey. Exclusion criteria were: (1) Older people with language barriers; (2) Older people with poor compliance; (3) Floating population in other provinces and cities. To encourage more respondents to participate in the survey, the research team provided food and some daily necessities to increase the enthusiasm of the respondents. Prior to the start of the survey, investigators met face-to-face with respondents, all of whom were informed of the purpose, meaning and process of the study and signed informed consent forms. At the same time, respondents were informed of their right to withdraw from the study at any time. A total of 1,255 respondents were recruited in the survey, of which 1,179 responded to the survey. After excluding those with missing key information and those who dropped out, a total of 1,065 respondents completed the questionnaire, with an effective response rate of 90.33%.

### Sociodemographic characteristics

The sociodemographic characteristics collected included sex (male, female), age (60–69 years, 70–79 years, ≥80 years), area of residence (urban, rural), marital status (single, married, others), and education level (no formal education, primary school, middle school, bachelor’s degree or above).

### Questionnaires

#### Health behavior

Smoking status, alcohol consumption and physical activity were among the health behavior variables assessed in this study using the Australian Active Survey (7, 22). This survey has good rest-retest reliability (23). Questions related to physical activity included whether respondents had engaged in physical activity or fitness activities in the previous 6 months, the type of exercise they most commonly performed, the average frequency and duration of weekly exercise, and the number of years of regular exercise.

Questions about smoking status included “Do you smoke now?” and “How often do you smoke?” Responses to the questions were assigned a coded value of 1 if the respondent was a current smoker, 2 if the respondent had quit smoking, and 3 if the respondent had never smoked. In terms of frequency, rarely or never smoking was assigned a value of 1, occasionally smoking was assigned a value of 2, and frequently smoking was assigned a value of 3.

The survey included questions such as “Do you drink alcohol?,” “how often do you drink alcohol?” and “Approximately how much alcohol do you consume per week?.” The answers to the questions were assigned a coded value of 1 if the respondent was currently drinking, 2 if the respondent had stopped drinking, and 3 if the respondent had never drunk. In terms of frequency, rarely or never drinking was assigned a value of 1, occasionally drinking was assigned a value of 2, and frequently drinking was assigned a value of 3. For alcohol consumption, based on measures from previous studies (24), the respondents were assigned a value of 1 if they drank standard amounts of alcohol >5 days per week; otherwise, the assigned value was 2.

#### Activities of daily living (ADL)

The ability to perform ADLS was assessed based on two measures: (1) performance of basic activities of daily living (BADLs) and (2) performance of instrumental activities of daily living(IADLs).

BADLs were assessed with the Barthel Index, a 10-item scale that has been shown to have high validity and reliability in the Chinese older adult population (25). The Barthel index includes items assessing eating, washing, grooming, dressing, bowel control, bladder control, toilet use, bed transfer, walking, and stairs climbing (26). The total score is 100, and the higher the score is, the more self-care that is needed in daily life (27). Scores of more than 60 points indicates good ADL function with basic self-care ability; scores of 40–60 indicate moderate dysfunction and the need for help with ADLs; scores of 20–40 indicate severe dysfunction and a marked dependence on others; and scores below 20 indicate complete disability and an inability to live on their own. The Cronbach’s α was 0.955 in this study.

IADLs were assessed in this study using the Functional Activities Questionnaire (FAQ) (28), which consists of 10 items to assess whether respondents are able to perform the following activities independently: shopping, cooking, using communication tools, cleaning, washing, purchasing medicines, using public transportation, managing finances, working, and hobbies. Each items is scored on a 3-point scale, from 0 (normal or not done, but can do) to 3 (completely dependent on others); a higher FAQ score indicates worse function (29, 30). The normal standard is less than 5 points; a score greater than or equal to 5 is an anomaly, indicating that respondents has difficulty being independent in their families and communities (31). The Cronbach’s α was 0.914 in this study.

#### Psychological distress

Psychological distress was measured using the Kessler Psychological Distress Scale (k10), which includes items regarding the frequency of experiencing related symptoms in the past 30 days. The scale consists of 10 items and measures psychological distress by examining depression (5 items) and anxiety (5 items) symptoms (32). Respondents are asked to answer questions based on a 5-point Likert scale. Each question is scored on a scale of 1 (almost never) to 5 (always), and the individual item scores are added to calculate the score. The final score range is 10–50, with higher scores indicating higher levels of psychological distress (33). Previous studies have proposed dichotomies of K10 scores, where scores of 22 and above indicate high to very high psychological distress, and scores of 22 and below indicate low to moderate psychological distress (33). The K10 scale has been widely used in academic and clinical research with strong reliability and validity across populations (32, 34). In this study, the Cronbach’s α was 0.931 for the whole scale.

#### Quality of life (QOL)

To assess QOL, the study used a universal scale developed by Life Europe, consisting of two subscales. The first subscale contains five dimensions: (1) mobility, (2) self-care, (3) daily living, (4) pain/discomfort, and (5) anxiety/depression. Each dimension contains only one question, for a total of five entries. The second subscale is a ‘Visual Analog Scale (VAS), where respondents rate their overall health for the day by drawing lines on the scale, with higher scores indicating better health (35). In this study, the EQ-VAS scores obtained with the EQ-5D scale and the VAS was used to easily reflect small changes in QOL. The application effect of the scale was previously shown to be significant in the Chinese population (36).The Cronbach’s α was 0.81 in this study.

#### Statistical analysis

SPSS 22.0 software (IBM Corporation, Armonk, NY, United States) and AMOS 23.0 software (IBM Corporation, Armonk, NY, United States) were used for statistical analysis. Descriptive analyses were performed to assess health behaviors, ADL performance, psychological distress, and QOL. Differences in QOL across socioeconomic demographic characteristics were analyzed using Fisher’s exact test and one-way analysis of variance. There were no missing values in this study.

According to the conceptual framework, structural equation modeling (SEM) was used to analyze the direct and indirect paths from health behavior to QOL and included two stages: (1) validation of the measurement model and (2) fitting of the structural model. The measurement model was constructed based on the CFA, the relationship between the observed and latent variables was described, the interrelations and covariates between the observed variables and the factor load were tested, and the model fit index was finally calculated (37). In this study, CFA was used to assess health behaviors, ADL, psychological distress, and QOL. The directionality of the relationship between latent and observed variables is determined by the structural model. To achieve an acceptable model fit, the structural model was fitted using maximum likelihood after model building. The model fitting results were good, with the following metrics: a χ2/df of less than 5, an approximate root-mean-square error (RMSEA) and standardized root-mean-square residual (SRMR) of less than 0.06, and a comparative fit index (CFI) with values greater than 0.90 (38). A bootstrap resampling procedure with 2000 samples and a bias corrected 95% confidence interval (BC 95% CI) were used to determine the statistical significance of the direct and indirect effects of each path. The total effect was calculated as the sum of direct and indirect effects and is mathematically expressed as: *c* = c0 + ab, where *c* = total effect, c0 = direct effect, and ab = indirect effect (39). A value of *p* < 0.05 indicates statistical significance.

## Results

### Sociodemographic characteristics and the distribution of QOL

The one-way ANOVA of QOL described by the sociodemographic features and the EQ-5D VAS is shown in Table 1. A total of 1,065 people completed the questionnaire, including 522 males (49.0%) and 543 females (51.0%). There were 466 (43.8%) people aged 60–69, 425 (39.9%) people aged 70–79, and 174 (16.3%) people aged 80 or older. There were 684 people (64.2%) registered in rural areas and 381 people (35.8%) registered in urban areas. Thirty-two people (3.0%) were single, 835 (78.4%) were married, and 198 (18.6%) were other. Thirty-four (3.2%) had a bachelor’s degree or above, 355 (33.3%) had a secondary education, 325 had a primary education (30.5%), and 351 had no education (33.0%). A total of 740 (69.5%) had never smoked, 193 (18.1%) were current smokers, and 132 (12.4%) were former smokers who had quit. There were 657 (61.7%) respondents who never drank alcohol, 275 (25.8%) who still drank alcohol, and 133 (12.5%) who used to drink alcohol and were currently abstainers. There were 467 people (43.8%) who regularly maintained physical exercise and 598 people (56.2%) who did not regularly maintain physical exercise. The ANOVA results showed that the differences in the EQ-5D VAS scores by age (*F* = 6.068, *p* < 0.01), education level (*F* = 3.090, *p* < 0.05), alcohol intake (*F* = 4.656, *p* < 0.05) and physical activity (*F* = 35.371, *p* < 0.01) ware statistically significant.

**Table 1:** Descriptive statistics of sociodemographic characteristics (*N* = 1,065).

Variables	EQ-5D VAS					
	*N*	(%)	Mean	SD	*F*	*P*
Sex						
Male	522	(49.0)	76.15	14.315	0.292	0.589
Female	543	(51.0)	75.68	13.960		
Age						
60-69 years	466	(43.8)	77.16	13.536	6.068	0.002^**^
70-79 years	425	(39.9)	75.80	13.837		
≥80 years	174	(16.3)	72.82	15.889		
Area of residence						
Urban	381	(35.8)	76.87	12.719	2.752	0.097
Rural	684	(64.2)	75.37	14.840		
Marital status						
Single	32	(3.0)	80.00	10.851	2.023	0.133
Married	835	(78.4)	76.02	13.841		
Others	198	(18.6)	74.76	15.645		
Educational level						
No formal education	351	(33.0)	74.47	14.413	3.090	0.026^*^
Primary school education	325	(30.5)	75.50	14.069		
Middle school education	355	(33.3)	77.48	13.696		
Bachelor’s degree or above	34	(3.2)	78.26	14.945		
Smoking status						
Never smoked	740	(69.5)	75.76	14.021	0.710	0.492
Currently smoke	193	(18.1)	76.94	13.914		
Ever smoked	132	(12.4)	75.23	15.063		
Alcohol consumption						
Never drank	657	(61.7)	75.42	14.129	4.656	0.010^*^
Currently drink	275	(25.8)	78.00	14.092		
Ever drank	133	(12.5)	73.98	13.827		
Physical activity						
Yes	467	(43.8)	78.78	13.115	35.371	<0.001^**^
No	598	(56.2)	73.67	14.496		

^*^p < 0.05, ^**^p < 0.01.

### Correlations between physical activity, cigarette and alcohol use, BADL performance, IADL performance, and QOL

Table 2 shows that physical activity and cigarette and alcohol use (*r* = −0.082, *p* < 0.01), BADL performance (*r* = −0.144, *p* < 0.01), IADL performance (*r* = −0.176, *p* < 0.05), psychological distress (*r* = −0.101, *p* < 0.01) and QOL (*r* = −0.143, *p* < 0.01) had a negative correlation; cigarette and alcohol use was positively correlated with psychological distress (*r* = 0.021, *p* < 0.01), BADL performance (*r* = 0.045, *p* < 0.05), IADL performance (*r* = 0.106, *p* < 0.01) and QOL (*r* = 0.087, *p* < 0.01). BADL was positively correlated with IADL performance (r = 0.719, *p* < 0.01), QOL(r = 0.771, p < 0.01) and psychological distress (*r* = 0.445, *p* < 0.01). IADL performance was positively correlated with psychological distress (*r* = 0.483, *p* < 0.01) and QOL (*r* = 0.658, *p* < 0.01). Psychological distress was positively correlated with QOL (*r* = 0.435, *p* < 0.01). The absolute value of skewness for each variable is within 2 and kurtosis is within 7, and the data can be considered approximately normally distributed.

**Table 2:** Correlations between the main variables.

Variables	*M*	SD	Skewness	Kurtosis	(1)	(2)	(3)	(4)	(5)	(6)
Physical activity (1)	14.10	1.844	−0.624	0.140	1.000					
Cigarette and alcohol use (2)	20.29	2.877	0.304	1.073	−0.082^**^	1.000				
BADL performance (3)	11.02	3.352	2.353	5.738	−0.144^**^	0.045^*^	1.000			
IADL performance (4)	13.01	5.322	2.449	6.565	−0.176^*^	0.106^**^	0.719^**^	1.000		
Psychological distress (5)	14.64	5.843	1.602	2.644	−0.101^**^	0.021^**^	0.445^**^	0.483^**^	1.000	
QOL (6)	75.91	14.130	−0.653	0.611	−0.143^**^	0.087^**^	0.771^**^	0.658^**^	0.435^**^	1.000

^**^p < 0.01, ^*^p < 0.05; M, mean; SD, standard deviation.

### Pathways from health behaviors to QOL

**Figure 2:**
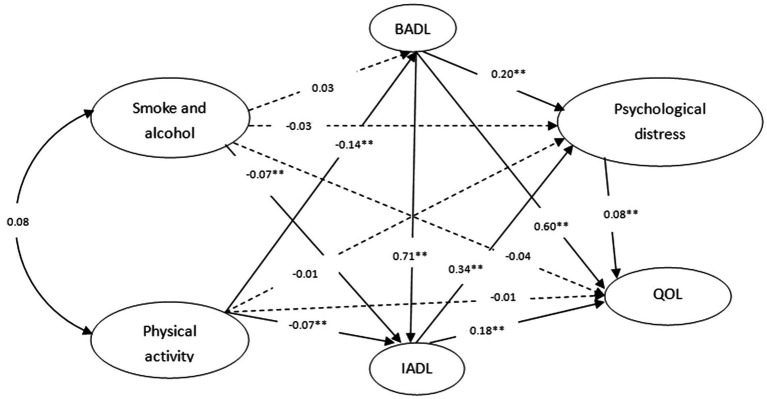
Standardized estimates of the pathways from cigarette and alcohol use and physical activity to QOL in the model. ^**^p < 0.01 Gray arrows refer to nonsignificant direct effects. Model fit: χ2/df = 2.897;RMSEA = 0.042; GFI = 0.914; AGFI = 0.906; CFI = 0.962.

All pathways passed bias-corrected Bootstrap tests (see Table 3), and standardized path estimates from smoking, alcohol consumption, and physical activity to QOL are shown in Figure 2, with dashed lines indicating non-significant direct effects. The standardized estimates of the direct, indirect, and total effects of cigarette and alcohol use and physical activity on QOL as well as the specific effects through BADL performance, IADL performance, and psychological distress are presented in Tables 4, 5. Cigarette and alcohol use had a nonsignificant direct effect (*β* =0.039) but significant indirect (*β* =0.037) and total (*β* =0.076) effects on QOL; physical activity had a nonsignificant direct effect (*β* = −0.013) but significant indirect (*β* = −0.124) and total (*β* = −0.137) effects on QOL; and cigarette and alcohol use significantly affected QOL through IADL performance (*β* = −0.012) explaining 15.8% of the total effects. Cigarette and alcohol use affected QOL through the sequential mediation of IADL performance and psychological distress (*β* = −0.002), explaining 2.6% of the total effects. Physical activity significantly impacted QOL through IADL performance (*β* = −0.013) and BADL performance (*β* = −0.084), explaining 9.5 and 61.3% of the total effects, respectively. Physical activity impacted QOL through the sequential mediation of IADL performance and psychological distress (*β* = −0.002), explaining 1.5% of the total effects. Physical activity influenced QOL through the sequential mediation of BADL performance and psychological distress (*β* = −0.002), explaining 1.5% of the total effects. Physical activity affected QOL through the sequential mediation of BADL performance and IADL performance (*β* = −0.018), explaining 13.1% of the total effects. Physical activity affected QOL through the sequential mediation of BADL performance, IADL performance and psychological distress (*β* = −0.003), explaining 2.2% of the total effects. The SEM approach indicated an acceptable model fit (χ2/df = 2.897, RMSEA = 0.042, GFI = 0.914; AGFI = 0.906; CFI = 0.962).

**Table 3:** Bootstrap test of bias correction for each action path.

Pathways	Estimate	Boot LLCI	Boot ULCI	*P*
BADL performance is ← Physical activity	−0.138	−0.184	−0.088	0.001^*^
BADL performance ← Cigarette and alcohol use	0.037	−0.019	0.085	0.219
IADL performance ← BADL performance	0.706	0.601	0.780	0.001^*^
IADL performance ← Physical activity	−0.072	−0.139	−0.043	0.001^*^
IADL performance ← Cigarette and alcohol use	0.071	0.030	0.121	0.002^*^
Psychological distress ← Physical activity	−0.005	−0.059	0.049	0.856
Psychological distress ← Cigarette and alcohol use	−0.033	−0.098	0.018	0.208
Psychological distress ← BADL performance	0.203	0.008	0.296	0.039^*^
Psychological distress ← IADL performance	0.342	0.268	0.540	0.001^*^
QOL ← BADL performance	0.597	0.449	0.709	0.001^*^
QOL ← IADL performance	0.179	0.088	0.350	0.003^*^
QOL ← Psychological distress	0.077	−0.008	0.155	0.044^*^
QOL ← Physical activity	−0.005	−0.054	0.041	0.811
QOL ← Cigarette and alcohol use	−0.036	−0.007	−0.073	0.097

^**^p < 0.01, ^*^p < 0.05; LL, low limit; CI, confidence interval; UL, upper limit.

**Table 4:** Standardized indirect path effects from health behavior to QOL (*N* = 1,065).

Pathways	Estimate	S.E.	*P*	BC 95% CI
Physical activity → IADL performance → QOL	−0.013^*^	0.003	0.005	(−0.027, −0.001)
Physical activity → BADL performance → QOL	−0.084^*^	0.009	0.001	(−0.146, −0.022)
Physical activity → Psychological distress → QOL	−0.001	0.001	0.42	(−0.026, 0.001)
Physical activity → IADL performance → Psychological distress → QOL	−0.002^*^	0.001	0.021	(−0.004, −0.001)
Physical activity → BADL performance → Psychological distress → QOL	−0.002^*^	0.001	0.031	(−0.003, −0.001)
Physical activity → BADL performance → IADL performance → QOL	−0.018^*^	0.003	0.004	(−0.026, −0.010)
Physical activity → BADL performance → IADL performance → Psychological distress → QOL	−0.003^*^	0.001	0.027	(−0.006, −0.001)
Cigarette and alcohol use → BADL performance → QOL	0.018	0.003	0.277	(0.013, 0.061)
Cigarette and alcohol use → BADL performance → Psychological distress → QOL	<0.001	0.001	0.110	(−0.001, 0.007)
Cigarette and alcohol use → BADL performance → IADL performance → QOL	0.004	0.001	0.178	(−0.023, 0.012)
Cigarette and alcohol use → BADL performance → IADL performance → Psychological distress →QOL	0.001	0.001	0.142	(−0.016, 0.015)
Cigarette and alcohol use → Psychological distress → QOL	- 0.002	0.001	0.242	(−0.013, 0.002)
Cigarette and alcohol use → IADL performance → QOL	−0.012^*^	0.001	0.04	(−0.021, −0.003)
Cigarette and alcohol use → IADL → Psychological distress → QOL	−0.002^*^	0.001	0.023	(−0.003, −0.001)

S.E., standard error; BC 95% CI, bias-corrected 95% confidence intervals, ^*^p < 0.05.

**Table 5:** Standardized direct, indirect and total effect of the variables on QOL (*N* = 1,065).

Variables	Standardized direct Effects	Standardized indirect Effects	Standardized total Effects
Cigarette and alcohol use	0.039	0.037^*^	0.076^*^
Physical activity	−0.013	−0.124^*^	−0.137^*^
BADL performance	0.603^*^	0.161^*^	0.764^*^
IADL performance	0.180^*^	0.026^*^	0.206^*^
Psychological distress	0.077^*^	–	0.077^*^

^*^p < 0.01.

## Discussion

This study developed a mediation model to elucidate the mechanisms by which health behaviors affect QOL through various mediating variables. The results showed that BADL performance, IADL performance and psychological distress had significant mediating effects on QOL in the health behavior intervention process. Moreover, the direct effect of health behavior interventions on QOL was relatively weak. These results confirm and expand upon previous research by proposing potential processes that connect health behaviors with QOL outcomes (40). Furthermore, this study uncovers the underlying mechanism through which health behaviors influence QOL and provides a theoretical foundation for enhancing the well-being of older adults. Additionally, it elucidates key mediators, thereby increasing the effectiveness of interventions aimed at improving and preventing poor QOL in older adults.

The mediating effect model shows that ADLs have multiple mediating effects on the relationship between health behaviors and QOL, and the incidence of ADL restriction in older adults is negatively correlated with reported results on QOL, which is consistent to some extent with previous studies (29). At the same time, several previous studies have concluded that limited ADL capacity in older adults with advanced disease is significantly associated with QOL (8, 41, 42). In addition, ADL performance, defined in other studies as “the ability to perform basic self-care activities in daily life,” is a predictor of QOL in older adults (43). However, these studies on both BADL and IADL ability, evaluated only a subset of ADLs and examined ADLs as an independent influence. Therefore, in this study, BADL performance and IADL performance were independently incorporated into the model as mediating variables, providing a broader perspective on the impact of health behaviors on QOL. According to the results of the study, compared with older adult respondents who did not smoke and drink, those who reported smoking and drinking and engaging in other adverse health behaviors experienced greater effects on BADL and IADL function, and the QOL report results were worse. At the same time, compared with older adult respondents who rarely or never performed physical exercise or participated in community activities, those who actively participated in physical exercise had relatively stronger ADL ability in terms of transferring, personal hygiene, walking and use of transportation, communication, housework and social cognition, and a higher ability to independently complete daily living activities, and did not need to rely on help from others. Their QOL was significantly better than the QOL of the respondents who never performed exercise or participated in community activities. This is consistent with the conclusions of previous studies (44). Furthermore, ADL not only mediates the relationship between healthy behavior and QOL directly, but also indirectly through psychological distress. According to previous research, older adults with negative health behaviors such as smoking, heavy drinking and lack of exercise have a significantly increased risk of declining physical health, leading to limitations in ADL levels, which leads to a long-term lack of confidence in solving problems, excessive dependence on others for help, and susceptibility to psychological distress such as anxiety and stress, which in turn affects QOL (45, 46). Therefore, we suggest that society and families pay more attention to older adult individuals’ ADL ability, for example, by providing convenient services such as special elevators, handrails or communication tools for older adult individuals, increasing the ability of older adult individuals to complete ADLs independently to improve their QOL, and providing them with communication and behavioral support.

According to the findings, psychological distress has a significant partial mediating role in the relationship between health behaviors and QOL. Previous studies have shown that the relative risk associated with smoking and psychological distress has been increasing over time, and the higher the smoking rate is, the more likely people are to develop psychological distress (21, 47). Another study showed that the association between little or no physical activity and psychological distress became stronger over time (21, 48, 49). In some studies, the association between alcohol consumption and psychological distress was relatively weak; however, this may have been due to different understandings of diverse notions of healthy behaviors and heterogeneous manipulation of different study designs. Health behaviors such as smoking, drinking and physical activity have been linked to psychological distress in most studies (21, 47–49). Psychological distress has a significant direct effect on the QOL of older adult individuals. This conclusion is consistent with previous research (50). Psychological distress should be taken seriously as a risk factor affecting the QOL of older adult individuals. High levels of fatigue, tension, anxiety and depression in older people can negatively affect QOL, and restlessness and frustration can easily lead to a decline in QOL (50, 51). Given the prevalence of complex health behavior (a combination of adverse and positive health behavior) and psychological distress in the older adult population, there is a clear need for society and families to pay more attention to the lifestyles and mental health of older people. For example, society should encourage older adult individuals to actively participate in community or collective activities, support them in carrying out moderate physical exercise by improving access to sports facilities and venues, and give sound advice to older adult individuals who smoke, drink, or engage in other behaviors to improve negative health behaviors to promote their health. At the same time, families should give more attention and encouragement to older people in life, boost their confidence to perform daily activities independently, and actively help them reduce psychological distress.

Surprisingly, health behaviors did not have a significant direct effect on the QOL of older adults in the present study model, while some Japanese and Singaporean scholars have reported negative associations between physical inactivity, work-related physical activity, and sedentary behavior and the QOL of older adults (52, 53). In addition, some Thai scholars have shown that unhealthy behaviors such as smoking and drinking not only directly affect the QOL of the older adult, but also indirectly intervene through chronic diseases (54). The results of this study also suggest that healthy behaviors have a significant direct effect on ADL performance and psychological distress. Older people with negative health behaviors, such as smoking and drinking, and a higher frequency of smoking and drinking, had more severe ADL limitations and psychological distress. Older adults who maintained positive health behaviors, such as participating in physical activity and community activities, had better ADL levels and mental health than those who engaged in little or no physical activity.

### Strengths and limitations

The strengths of this study include the following: First, the use of regionally representative population data to study the association between health behaviors and QOLs among older adults in Xuzhou provides findings with general applicability to China and even other Asian countries. Second, the commonly accepted health behavior scales, the K10, the Barthel Index, and the EQ-5D-VAS scale and criteria, were used to measure health behavior, psychological distress, ADL performance and QOL. Third, the association between health behaviors and QOL was analyzed by means of ADL and multiple mediation analysis of psychological distress.

Despite these advantages, there are some notable limitations. First, health behaviors are a combination of different behavioral patterns, and the study did not capture all relevant behaviors; for example, dietary habits such as fruit and vegetable consumption, sleep quality, and sedentary duration were not included. Second, the EQ-VAS was used to measure QOL in this study, but the EQ-VAS is based on self-assessments of individual respondents, which may have led to some interference in assessing QOL for the population as a whole. Third, this study was cross-sectional, which made it difficult to comprehensively monitor changes in ADLs and psychological distress over time in older adults. Although the current findings suggest a relationship between health behaviors and QOL and the mediating role of ADLs and psychological distress, the nature of this study precludes causality. Further experimental studies are needed to better determine whether mediating variables such as ADL performance and psychological distress have a bidirectional relationship with health behaviors and QOL. Finally, the respondents were surveyed on a scientific scale, but the results were obtained based on the respondents’ memories, which may be subject to recall bias.

## Conclusion

In conclusion, the study found the following conclusions: (1) There is a relationship between health behaviors and QOL, and the relationship may be bidirectional: positive health behaviors lead to better QOL, while poor health behaviors lead to worse QOL. (2) Health behaviors could indirectly affect QOL through ADL performance. (3) Psychological distress had multiple mediating effects, which could mediate the association between health behaviors and QOL directly and indirectly through ADL intervention. Families and society should pay attention to incentive strategies to encourage older adult individuals to avoid negative health behaviors and carry out positive health behaviors related to improving the QOL, improving the confidence of the older adult to independently complete the activities of daily living, and reducing psychological distress to achieve better results in the process of improving the QOL of older adult individuals.

## Data availability statement

The raw data supporting the conclusions of this article will be made available by the authors, without undue reservation.

## Author contributions

YW: Data curation, Investigation, Writing – original draft. JX: Methodology, Supervision, Writing – original draft. YG: Investigation, Writing – original draft. JZ: Conceptualization, Funding acquisition, Methodology, Writing – review & editing.
